# A polymorphism in the haptoglobin, haptoglobin related protein locus is associated with risk of human sleeping sickness within Cameroonian populations

**DOI:** 10.1371/journal.pntd.0005979

**Published:** 2017-10-27

**Authors:** Elvis Ofon, Harry Noyes, Julius Mulindwa, Hamidou Ilboudo, Martin Simuunza, Vincent Ebo’o, Flobert Njiokou, Mathurin Koffi, Bruno Bucheton, Pythagore Fogue, Christiane Hertz-Fowler, Annette MacLeod, Gustave Simo, for the TrypanoGEN Research Group, as members of The H3Africa Consortium

**Affiliations:** 1 Molecular Parasitology & Entomology Unit, Department of Biochemistry, Faculty of Science, University of Dschang, Dschang, Cameroon; 2 Centre for Genomic Research, University of Liverpool, Liverpool, United Kingdom; 3 Department of Biochemistry, CONAS, Makerere University, Kampala, Uganda; 4 Unité de recherche sur les bases biologiques de la lutte intégrée, Centre International de Recherche-Développement sur l’Elevage en zone Subhumide (CIRDES), Bobo-Dioulasso, Burkina Faso; 5 Department of Disease Control, School of Veterinary Medicine, University of Zambia, Lusaka, Zambia; 6 National Sleeping sickness control Program of Cameroon, Ministry of Public Health, Yaoundé, Cameroon; 7 Laboratory of Molecular Biology, Department of Animal Biology, Faculty of Science, University of Yaoundé 1, Yaounde, Cameroon; 8 Laboratoire des interactions Hôte-Microorganismes-Environnement et Evolution, Unité de Formation et de Recherche Environnement, Université Jean Lorougnon Guédé, Daloa, Côte d’Ivoire; 9 Institut de Recherche pour le Développement (IRD), UMR INTERTRYP IRD-CIRAD, Campus international de Baillarguet, Montpellier, France; 10 Programme National de Lutte contre la Trypanosomose Humaine Africaine, Conakry, Guinea; 11 Centre for Genomic Research, University of Liverpool, Liverpool, United Kingdom; 12 Institute of Biodiversity, Animal Health and Comparative Medicine, University of Glasgow, Garscube Estate, Glasgow, United Kingdom; Hunter College, CUNY, UNITED STATES

## Abstract

**Background:**

Human African Trypanosomiasis (HAT) is a neglected disease targeted for elimination as a public health problem by 2020. Elimination requires a better understanding of the epidemiology and clinical evolution of HAT. In addition to the classical clinical evolution of HAT, asymptomatic carriers and spontaneous cure have been reported in West Africa. A genetic component to human susceptibility to HAT has been suggested to explain these newly observed responses to infection. In order to test for genetic associations with infection response, genetic polymorphism in 17 genes were tested (*APOL1*, *IL1B*, *IL4*, *IL4R*, *IL6*, *IL8*, *IL12B*, *IL12RB1*, *IL10*, *TNFA*, *INFG*, *MIF*, *HLA-G*, *HLA-A*, *HP*, *HPR and CFH*).

**Methodology:**

A case-control study was performed on 180 blood samples collected from 56 cases and 124 controls from Cameroon. DNA was extracted from blood samples. After quality control, 25 samples (24 controls and 1 case) were eliminated. The genotyping undertaken on 155 individuals including 55 cases and 100 controls were investigated at 96 loci (88 SNPs and 8 indels) located on 17 genes. Associations between these loci and HAT were estimated via a case-control association test.

**Results:**

Analyses of 64 SNPs and 4 indels out of 96 identified in the selected genes reveal that the minor allele (T) of rs8062041 in haptoglobin (*HP*) appeared to be protective against HAT (p = 0.0002395, OR 0.359 (CI_95_ [0.204–0.6319])); indicating higher frequency in cases compared to controls. This minor allele with adjusted *p* value of 0.0163 is associated with a lower risk (protective effect) of developing sleeping sickness.

**Conclusion:**

The haptoglobin related protein *HPR* and *HP* are tightly linked and both are duplicated in some people and may lead to higher activity. This increased production could be responsible of the protection associated with rs8062041 even though this SNP is within HP.

## Author summary

Human African trypanosomiasis (HAT) or sleeping sickness is a neglected tropical disease targeted for elimination by 2020. This elimination requires a better understanding of the epidemiology and clinical evolution of this disease. Beside the classical clinical evolution, asymptomatic carriers, seropositive and spontaneous cure of infected persons have been reported in West Africa. Arguments in favor of human genetic susceptibility to HAT have been raised to explain this variability in clinical presentation. This study investigated the genetic polymorphism of 17 genes between controls and sleeping sickness patients in Southern Cameroon in order to improve our knowledge of human susceptibility to trypanosome infections. We identified single nucleotide polymorphisms and indels in 17 selected genes involved in immune responses and carried out a case-control candidate gene association study and demonstrated differences between variants associated with the disease. From these genes, only haptoglobin (*HP*) at the SNP rs8062041 was found to have polymorphisms which were strongly associated with trypanosomiasis. The minor allele (T) at this SNP position appeared to be protective against HAT (p = 0.0002395, OR 0.359 (CI_95_ [0.204–0.6319])) reducing the risk of developing disease approximately threefold. The haptoglobin related protein (*HPR*) is adjacent to *HP* and is a component of the Trypanolytic factor that kills trypanosomes. The *HP* and *HPR* locus is duplicated in some people. The rs8062041 variant may be associated with this duplication and it is possible that increased production of HPR is the cause of the protection associated with rs8062041. The results reported here will contribute to the knowledge of the role of human genetics in disease progression, and thus lead to the identification of novel biomarkers which could involve development of new diagnostics, treatments and intervention strategies.

## Introduction

Human African Trypanosomiasis (HAT) or sleeping sickness is a lethal neglected tropical disease responsible for severe morbidity and economic losses in areas where it occurs [1]. HAT is caused by subspecies of *Trypanosoma* that are transmitted to humans through the bites of hematophagous flies of the genus *Glossina*, commonly known as tsetse flies. HAT exists in two forms: the acute form due to *Trypanosoma brucei rhodesiense*, which occurs in East Africa, and the chronic form due to *T*. *b*. *gambiense*, which is found in West and Central Africa. More than 98% of the currently reported cases belong to the chronic form. About 65 million people are estimated to be at risk of HAT and the current number of HAT cases is below 20,000 pa [2]. Control efforts undertaken during the last decades have reduced considerably the number of cases and 3,796 new cases were reported to WHO in 2014 [2]. With the success of these control efforts, HAT has been included in the WHO roadmap of neglected tropical diseases which are targeted for elimination as a public health problem by 2020. For effective control, it is important to gain a better understanding of the clinical evolution of the disease. Previously, HAT was classically considered to be fatal if untreated. During the last decades, a range of clinical presentations of *T*. *b*. *gambiense* HAT have been reported including asymptomatic carriers and spontaneous cure without treatment [3]. One hypothesis for the diversity of clinical outcomes that occur during infections due to *T*. *b*. *gambiense* is that it is due to human genetic variability. Previous investigations on genes such as *HLA-A*, *HP*, *CFH*, *IL1B*, *IL12B*, *IL12RB1*, *IL4R* and *HPR* [4, 5, 6, 7, 8, 9, 10, 11, 12] revealed associations between the polymorphisms in some of these genes with infectious diseases including HIV, viral hepatitis, malaria and tuberculosis. In HAT, polymorphisms in sequence or expression of genes involved in immune response such as *APOL1*, *IL4*, *IL6*, *IL10*, *IL8*, *TNFA*, *HLA-G*, *MIF*, *HPR* and *INFG* have been investigated for their association with the outcome of *T*. *b*. *gambiense* infections [13, 14, 15, 16, 17, 18, 19, 20, 21, 1]. These investigations found associations between some polymorphisms in genes and the risk of developing HAT. For instance, Courtin *et al*. [14–16] have shown that polymorphisms in *IL6*, *IL10* and *HLA-G* were associated with a protective effect against HAT. In addition, a protective effect has been observed *in vitro* and *in vivo* of the G2 allele of *APOL1* against infections due to *T*. *b*. *rhodesiense* [22]. In Guinea, the G1 allele of APOL1 was found to be associated with protection of asymptomatic individuals against development of active disease [22]. Despite these observations, the relationship between genetic polymorphisms and susceptibility to HAT is still not well understood and further investigations on populations of more HAT foci are required.

To improve our knowledge of the genetic determinants that could play important roles during infections due to *T*. *b*. *gambiense*, seventeen genes were selected in this study and their polymorphisms were investigated within populations of three sleeping sickness foci of the forest region of Cameroon.

### Study area and study population

**Fig 1:**
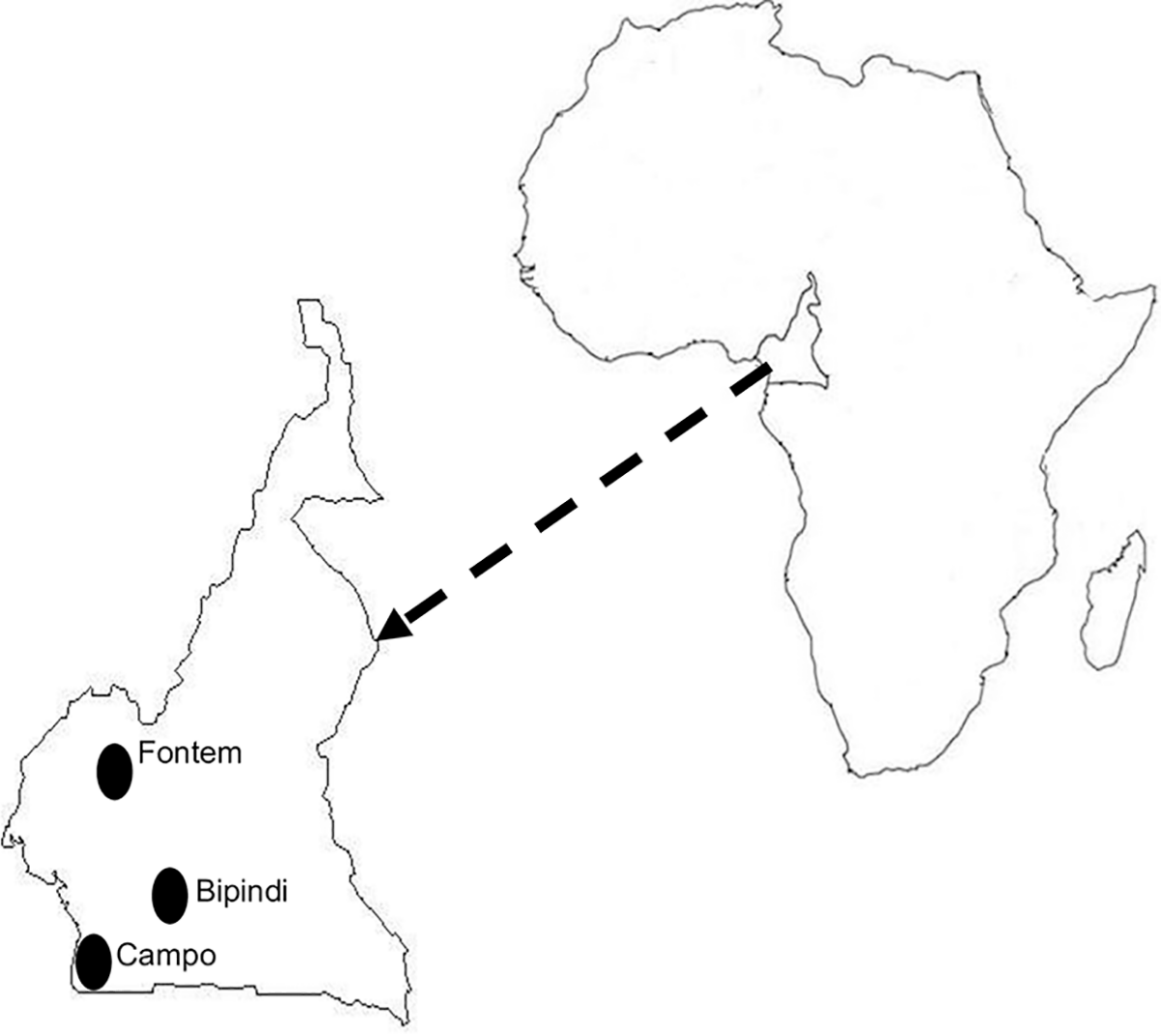
HAT foci where samples were collected.

The study was conducted in three active sleeping sickness foci of the forest region of Southern Cameroon (Fig 1). The Cameroonian population is made up of more than 240 ethnic groups that can be grouped into Bantu (e.g.: Beti, Bassa, Bakundu, Maka, Douala, Pygmie), Semi Bantu (e.g.: Bamileke, Gbaya, Bamoun, Tika) and Sudano-Sao (e.g.: Fulbe, Mafa, Toupouri, Shoa-Arabs, Moundang, Massa, Mousgoum). The composition varies considerably between HAT foci and even within the same HAT focus. The three HAT foci where this study was undertaken were Bipindi and Campo in the Southern region and Fontem in the South-west region of Cameroon.

The Campo focus (2°82'00"N, 9°85'20"E) is located in the tropical forest and extends from the Atlantic coast along the Ntem river which delimits the Cameroon–Equatorial Guinea border. It is a hypo-endemic focus with no history of epidemic outbreaks [23] and a cumulative number of 98 cases were detected between 1998 and 2013. The main source of livelihood for the inhabitants of the Campo focus is agriculture, fishing and hunting. It is a cosmopolitan area with several ethnic groups including mainly the Iyassa, Kwasse, Maabi, Mvae and Ngoumba, most of whom are Bantu speaking. Other minor ethnic groups are semi Bantus and Sao-Sudanese and can be found at Campo for administrative and socioeconomic purposes.

The Bipindi HAT focus (3°82'00"N, 10°82'20"E) is located at about 75 km from the Atlantic coast in the South of Cameroon. It is an old HAT focus that has been known since 1920. During the last two decades, the Bipindi focus was among the most active HAT foci of Cameroon with around 83 HAT cases diagnosed from 1999 to 2011 [24]. About 95% of the inhabitants of the Bipindi HAT focus are Bantu speaking and belong to ethnic groups such as Ngoumba, Nti, Fan and Pygmies. The remaining 5% of inhabitants (semi Bantus and Sao-Sudanese) are there for administrative and socioeconomic purposes. The main livelihood for people in this focus is hunting, farming and seasonal harvesting of fruits.

The Fontem focus (5°40’00”N, 9°55’00”E) is located in the South-West Region of Cameroon where HAT has been known to occur since 1949 [25]. The Fontem focus was previously among the most active HAT foci of Cameroon [26], but in recent decades, it has become hypoendemic with about 8 patients detected among 16,000 persons examined between 1998 and 2007 [27]. In this focus, the Mundani, Bamoua and Bangwa are the major ethnics groups. Other minor ethnic groups such as Banyangue and Bamileke are also found.

## Materials and methods

### Sample collection

The blood samples were collected during medical surveys performed jointly with the national sleeping sickness control program of Cameroon. The sampling was done at Campo in 2014 and for Bipindi and Fontem, in 2015. During these surveys, all inhabitants were screened with CATT test [28] on whole blood. All inhabitants with positive CATT test were subjected to CATT dilution on plasma and each inhabitant positive on a CATT dilution ≥1/8 was subjected to parasitological examination (capillary tube centrifugation (CTC) [29] and minianion exchange centrifugation technique (mAECT)) [30]. For all inhabitants with CATT dilution ≥1/8 and negative for all parasitological tests, 90μl of plasma were spotted on a Whatman paper disc (divided in four equal parts with each bearing a spot of 30μl) that was sent to CIRDES in Burkina Faso for the trypanolysis test [31]. Beside the CTC and mAECT, lymph node aspiration followed by a microscopic examination was performed to search for trypanosomes in all individuals showing enlarged lymph nodes. A new HAT case was defined as an inhabitant in whom trypanosomes were seen by at least one parasitological method. Beside these new HAT cases, old HAT cases were also resampled. Old HAT cases were residents in whom trypanosomes had been previously seen on at least one parasitological test after passive or active case detection. Old HAT cases were only included in this study if the information regarding the clinical status, the CATT test and all parasitological tests were available in hospital records. A control was considered as any individual negative for the CATT test, all parasitological tests including CTC, mAECT and lymph node examination and when possible the trypanolysis test.

With these sampling criteria, 5ml of peripheral venous blood samples were collected from cases and controls into EDTA coated tubes. In the field, the tubes were mixed gently and stored at 4°C in an electric cooler before being transported to the laboratory.

### Ethics statement

The protocol of this study was approved by the Ethical Committee of the Ministry of Public Health of Cameroon reference number N°2013/11/364/L/CNERSH/SP of 21 November 2013. The local administrative and traditional authorities of each HAT focus were also informed and gave their approval. Subsequently, the review board (LAMAS) of Laboratory of Microbiology and Anti-microbial substances of the Department of Biochemistry of the Faculty of Science of the University of Dschang gave their approval. All adult subjects provided informed consent, and a parent or guardian of any child participant below 18 years old provided informed consent on their behalf. Each informed consent was written because all individuals enrolled in this study gave their approval by signing an informed consent form and a Certificate of Confidentiality. During analyzes, data of each subject were anonymized.

### DNA extraction

Blood samples were centrifuged at 5000rpm for 3 minutes and the buffy coat was collected. Genomic DNA was extracted from the Buffy-coat with the QIAamp DNA Blood Midi/Maxi kit (Qiagen) according to the manufacturer's instructions. The DNA was eluted with 200μl of elution buffer and stored at -20°C until use.

### Power calculation

Power calculations were undertaken using the genetics analysis package gap in r [32]

### Selection of candidate genes

The choices of candidate genes were based on previous observations. The cytokines *IL4*, *IL6*, *IL10*, *IL8*, *INFG*, *TNFA*, *HP*, *HPR* and *MHC* gene *HLA-G* were selected because they have been previously associated with HAT [14–16, 20, 33, 34, 35, 36, 37]. In addition, two genes for factors involved in the lysis of trypanosomes, *APOL1* and haptoglobin-related protein (*HPR*) were also included [17, 18, 19]. Five further genes that had previously been reported to play an important role in the susceptibility to other infectious diseases were selected: Human Leukocytes Antigen A (*HLA-A*) [38, 4, 39], IL1B [10], Complement factor H (*CFH*) [6, 10], *IL12B* and *IL12RB1* [5, 11] and Macrophage migration inhibitory factor (*MIF*) [40, 41, 42] genes were also included.

### SNPs and INDELs selection

Most SNPs and indels for testing were selected after a Linkage scan (r² = 0.5) and quality control with Plink version 1.9 [43] using whole genome sequencing data. These data were obtained from a merged dataset between the African populations data from the 1000 Genomes Project combined with low fold coverage (8-10x) whole genome shotgun data generated from 230 residents living in regions (DRC, Guinea Conakry, Ivory Coast and Uganda, European Genome Archive A accession number) where trypanosomiasis is endemic [44]. The 88 SNPs and 8 indels loci were selected by two strategies: 1) by linkage scan of SNPs and indels (r^2^ < 0.5) across the gene; 2) by selection of SNPs and indels with published associations with HAT. Linked SNPs were identified for *IL6*, *IL4*, *IL8*, *IFNG* and *HLA-G* genes. For *APOL1*, *HPR*, *HP*, *HLA-A*, *IL1B*, *IL12B*, *IL12RB1*, *IL4R*, *CFH*, *IL10*, *MIF* and *TNFA* genes, individual published SNPs and indels were identified and selected based on literature searches.

### Genotyping

Samples which had low DNA concentration or did not satisfy the quality control criteria were excluded prior to genotyping. Genotyping was performed by two commercial service providers: 1) “Plateforme Genome Transcriptome” at INRA of Bordeaux in France; 2) LGC Genomics Hoddesden, UK with approximately 1μg of genomic DNA per sample.

At INRA, genotyping was carried out with a Multiplex design (two sets of 40 SNPs or indels) using Assay Design Suite v2.0 (Agena Biosciences). For each SNP and indel, the genotyping was done with the iPLEX Gold genotyping kit (Agena Biosciences) for the Mass-Array iPLEX genotyping assay according to the manufacturer’s instructions. Products were detected on a Mass-Array mass spectrophotometer and data were obtained in real time with Mass-Array RT software (Agena Biosciences). SNP clustering and validation was carried out with Typer 4.0 software (Agena Biosciences). A summary of the candidate genes, and SNPs and indels is shown in the supplementary data S1 Table. Some SNPs and indels that failed genotyping at INRA and some additional SNPs and indels were genotyped at LGC Genomics, Hoddesden, UK where SNPs and indels were genotyped using the PCR based KASP assay [45].

### Analysis

This was a case-control study where no familial controls were collected during sampling. The raw genotypic data were converted to PLINK format and quality control (QC) procedures implemented using the PLINK v1.9 package [43]. The Spearman Chi-square test was used to compare frequencies of observed and expected genotypes under Hardy–Weinberg equilibrium (HWE) and LD using R/Rstudio version 3.3.2 (2016-10-31)—‘Sincere Pumpkin Patch’ and Plink [43]. After quality control and filtering, poorly performing SNP loci with missing genotypes (≥10) and samples with missing loci (≥4) were removed. In addition, all loci with a MAF below 1% or a HWE *P* value < 1 × 10^−4^ were removed. SNP in linkage with adjacent SNP (r² > 0.5) were also pruned. These filters are as described by Anderson *et al*. [46] to minimize the influence of genotype-calling artifacts in a candidate gene study. The association between individual SNPs and indels within genes and HAT were tested using the Fisher exact test with Plink v1.9 software. Results were adjusted for multiple testing by Bonferroni correction. To show significant association during multiple tests, a single marker (SNP) must show, after Bonferroni correction, an alpha value (obtained P value before correction/number of SNPs analyzed) below 0.000746 (0.05/68). The Bonferroni correction assumes that each of the statistical tests is independent; however, this is not always true due to the possibility of linkage disequilibrium among the SNPs. In instances where the assumption is not true, the correction is often too strict, leading potentially to false negatives. A less stringent correction for multiple testing was also employed. The Benjamini-Hochberg false discovery rate (FDR) estimates the proportion of significant results (*P* < 0.05) when the Bonferroni correction considers them as false positives [47, 48]. F_ST_ is a measure of differences between populations. The analysis of F_ST_ was run to check for significant allele frequency difference between the cases and controls while Principal Component Analysis (PCA) was used to check for population stratification that might confound the analysis using Plink [43].

### Accession number

European Genome Archive A accession number: EGAS00001002602.

## Results

### Study design and population

This study was one of six studies of populations of HAT endemic areas in Cameroon, Cote d’Ivoire, Guinea, DRC, Malawi and Uganda. The studies were designed to have 80% power to detect odds ratios (OR) >2 for loci with disease allele frequencies of 0.15–0.65 and 100 cases and 100 controls with the 96 loci genotyped.

Overall, 216 individuals were included in this study: 56 (25.93%) HAT patients and 160 (74.07%) controls. The 216 individuals belonged to 22 different ethnic groups. The mean age (range) of HAT cases was 44.94 (15–82) years, while that of controls was 37.08 (9–86). The overall sex ratio (male/female) was 1.02 (109/107), with HAT cases being 0.75 (24/32) and controls 1.12 (84/75). Given that only 56 cases were available from our study area, the power of this study was reduced and it had 80% power to detect an OR >3 with disease allele frequencies of 0.1–0.45 with the 96 loci genotyped.

One hundred and eighty (56 HAT cases and 124 controls) of the 216 samples were sent for genotyping. After DNA quantification and quality control on each of these 180 samples, 25 were excluded from genotyping. 155 samples were genotyped: 55 (34.48%) HAT cases and 100 (64.52%) controls.

### Genes and SNPs selected and genotyped

96 loci containing 88 SNPs and 8 indels were tested from 17 candidate genes. The number of SNPs and indels analyzed varied considerably (from 1 to 18) between genes (Table 1). The highest number of 18 SNPs and indels was observed for *HLA-G* and the lowest number of one SNP for *IL10*, *IL1B* and *CFH*. However, it is important to point out that for *APOL1* (three SNPs), *CFH*, *TNFA*, *HLA-A* and *IL10*, the SNPs considered here are only those that have been already reported in the literature. Of the 88 SNPs and 8 indels used in this study, 24 SNPs and 4 indels (with 8 removed for MAF ≤1%, 7 for missing loci ≥10%, 5 with HWE P-values <1 x 10^−4^ and 8 for linkage at r² ≥ 0.5) of them were excluded during quality control which excluded one gene (*HLA-A*) completely. Four samples were also excluded during quality control due to missing individual data ≥4%. For subsequent analyses, 69.29% of loci including 64 SNPs and 4 indels from 16 genes and 151 (97.42%) samples will be considered for association analysis (Table 1).

**Table 1:** Number of SNPs and indels identified and selected for each gene.

Chromosome	Gene	Number of SNPs and indels identified and selected	Number SNPs and indels retained*
1	*IL10*	1	1
1	*CFH*	1	1
2	*IL1B*	1	1
4	*IL8*	6	5
5	*IL4*	16*	13*
5	*IL12B*	2	2
6	*HLAG*	18*	9*
6	*TNFA*	3*	2
6	*HLAA*	3	-
7	*IL6*	12	10
12	*IFNG*	10	10
16	*HP*	2	2
16	*HPR*	3	2
16	*IL4R*	1	1
19	*IL12RB1*	2	1
22	*MIF*	9	3
22	*APOL1*	6*	5*

* Genes with indels loci genotyped and retained after QC for analysis

The principal components (S1 Fig: supplementary data) and F_ST_ values (S2 Table: supplementary data) analysis showed that cases and controls were evenly dispersed (homogenous and samples did not cluster by phenotype); indicating that the population and subpopulation structure is not the driving force in our observations.

### Genotyping results

Alleles for the sixteen genes and 64 SNPs and 4 indels analyzed were all in Hardy–Weinberg equilibrium (S2 and S3 Tables: supplementary data); suggesting random genetic exchange within the studied populations. The MAF varied considerably across SNP and indel (S2 Table: supplementary data) with the lowest MAF at rs11575934 in *IL12RB1* (MAF = 0.0067) and the highest value at rs371194629 in *HLA-G* (MAF = 0.5).

**Fig 2:**
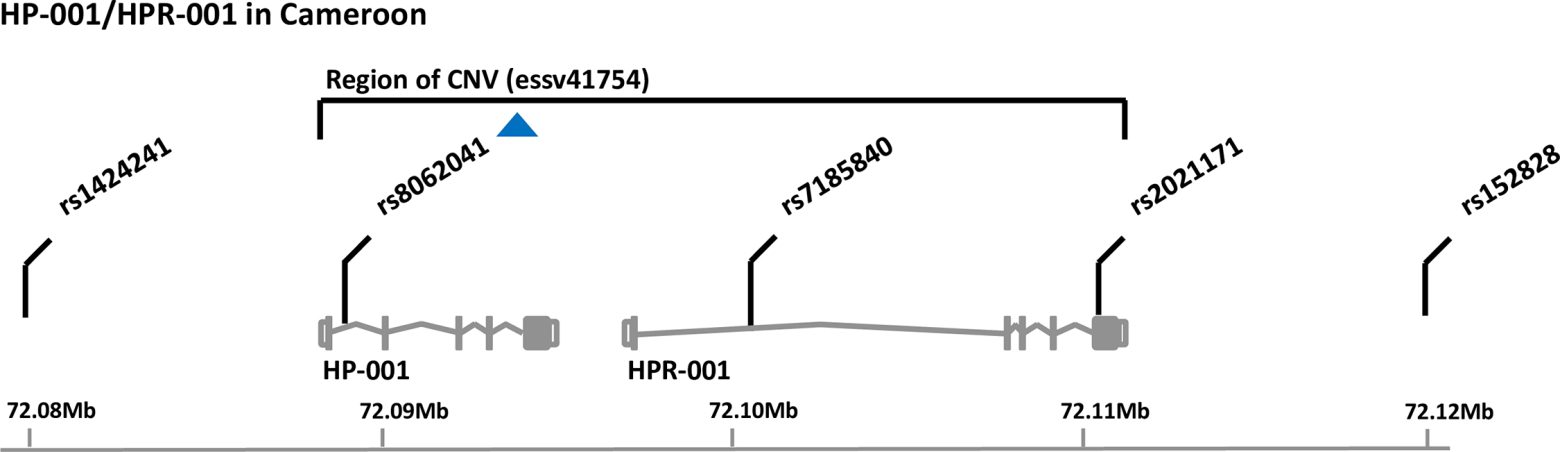
Locations of SNPs genotyped on HP/HPR region.

The minor allele (T) of rs8062041 in *HP* appeared to be protective against HAT (p = 0.00024). An odds ratio (OR) of 0.359 (CI_95_ [0.20–0.63]) indicated low frequencies in cases compared to controls. This SNP is located in a copy number variation (CNV) essv41754 that spans both HP and HPR (Fig 2).

In addition, the minor alleles of *IL4* and *HLA-G* also appeared protective (IL4: C rs2070874, uncorrected p = 0.047: and *HLA-G*: G rs1233330, uncorrected p = 0.011). The OR of 0.62 (CI_95_ [0.38–1.01]) for rs2070874-IL4 C and 0.2754 (CI_95_ [0.093–0.81]) for rs1233330 *HLA-G* also indicated low frequencies of the major allele in cases compared to controls. However, for *HLA-G*, the minor allele (A) of SNP rs17875389 had a higher frequency in cases than controls (p = 0.042). The OR of 2.29 (CI_95_ [0.97–5.39]) suggests that the A allele may increase the risk of developing HAT.

Of the 64 SNPs and 4 indels considered here, only four (SNP) of them belonging to three genes were associated with the development of HAT before Bonferroni correction (Table 2). After Bonferroni correction only one SNP (rs8062041 T/C) in *HP* was associated with HAT. The odds ratio of 0.359 suggests that the minor allele has a protective effect within the Cameroonian population with 95.3% (FDR) chance of this locus being associated with HAT (Table 2).

**Table 2:** Loci with significant associations with HAT.

CHR	Gene	SNP	BP	Allele	F_A	F_U	Nominal P	OR	Hwe-P	MAF	BONF	FDR_ BH
6	HLA-G	rs1233330	29799103	A*	0.04167	0.1364	0.011	0.275	0.0157	0.1054	0.67	0.3632
				G**								
6		rs17875389	29794484	G*	0.1176	0.055	0.042	2.291	1	0.07616	1	0.6708
				A**								
16	HP	rs8062041	72088964	T*	0.2959	0.4948	0.0002	0.359	1	0.4338	0.016	0.01629
				C**								
5	IL4	rs2070874	132009710	T*	0.3529	0.47	0.0071	0.615	0.5463	0.4305	1	0.722
				C**								

SNP: single nucleotide polymorphism, BP base-pair location

* minor allele

** major allele; F_A & F_U frequency of the minor allele in cases and controls respectively; Nominal P unadjusted asymptotic probability value; OR odds ratio; HWE-P Hardy-Weinberg equilibrium p value for unaffected individuals; BONF Bonferroni adjusted asymptotic p value, FDR_BH = False Discovery Rate Benjamin-Hochberg, MAF Minor allele frequency, CHR: Chromosome.

For the three remaining SNPs where the association was not significant after Bonferroni correction, our results show that the allele frequencies in cases and controls were not the same (Table 2) and that there is some possibility of an association with disease. FDR_BH is the probability of falsely rejecting the null hypothesis that allele frequencies are the same in cases and controls. rs1233330 and rs17875389 in *HLA-G* had FDR_BH values of 0.36 and 0.67 respectively; suggesting that there are 64% and 33% probabilities of an association between SNPs at these loci with HAT. For rs2070874 in *IL4*, the FDR_BH value of 0.722 suggests 27.8% chance of an association with HAT.

For the other genes (*APOL1*, *IL4*, *IL6*, *IL10*, *IL8*, *TNFA* and *INFG*) involved in immune response that have been previously investigated in HAT, our results revealed no statistical association with the disease within the Cameroonian population (S1 Table). No association was also observed with SNP and indel of *APOL1* and all SNPs of *HLA-A*, *IL1B*, *IL12B*, *CFH*, *IL12RB1*, *IL4R and MIF* previously associated with the susceptibility to other infectious diseases (S2 Table: supplementary data).

## Discussion

In this study we obtain good quality genotype data for a total of 64 SNPs and 4 indels in 16 genes to investigate associations with trypanosomiasis. Of these genes selected on the basis of their association with HAT or other infectious diseases, most were not statistically associated with HAT in Southern Cameroon.

The most important result of this study is the observation that the T allele of SNP rs8062041-*HP* with a p-value of 0.00024 (Bonferonni corrected p = 0.015) and an OR of 0.36 is associated with a lower risk (protective effect) of developing sleeping sickness. This SNP lies within intron 1–2 of *HP* of the CNV essv41754 that spans both *HP* and *HPR* transcripts (Fig 2). Although the biological significance of this CNV is not well understood, it is important to point out that *HP* and *HPR* have some biological similarities. Haptoglobin is involved in the scavenging of haem from lysed red blood cells. Trypanosome infections induce extensive lysis of red cells releasing haem which is scavenged by HP. In mice, the expression of the haptoglobin receptor (*Cd163*) on macrophages declines dramatically after infection with *T*. *congolense* [49] and is the earliest indicator of infection. HPR also binds haem but is not cleared from circulation after haemolysis. However, *HPR* is of particular interest because it plays a prominent role in the innate resistance of humans to most *Trypanosoma* species [50]. This innate resistance is linked to trypanosome lytic factors 1 and 2 (TLF1, TLF2) which are bound to a minor subclass of high-density lipoprotein (HDL) [51]. Both factors harbor APOL1, which is the trypanolytic component [52], and HPR which facilitates the uptake of APOL1 via trypanosome haptoglobin–hemoglobin receptors (HpHbR).

Interestingly, rs8062041-*HP* (T/C) is located on chromosome 16 (16q22.2) in the CNV essv41754 that spans both HP and HPR (Fig 2). Such genomic structural variants involving *HP/HPR* duplication have been reported with higher frequency in people of African descent [53, 7]. For instance, *HP* and *HPR* have been reported in 29 independent studies listed in the Database of Genome Variants [54]. *T*. *b*. *gambiense* protects itself against killing by APOL1 by reducing the abundance and affinity of the receptor for HPR [55]. If rs8062041, located in the CNV essv41754 spanning HP and HPR is correlated with CNV genotype, then an increase in HPR expression could drive increased uptake of APOL1 and parasite killing. As in other diseases such as heart disease, cancer, malaria and Crohn’s disease, polymorphism in *HP* could also have direct biological significance in HAT. Polymorphism in the haptoglobin gene may be associated with reduced cholesterol levels in the blood [56] and since cholesterol is specifically taken up by trypanosomes as a nutrient, any reduction in cholesterol might restrict parasite growth rate. There are numerous variants of *HP*, some of which may have arisen from gene conversion from *HPR* exons [56]. Single SNP tag these variants poorly (max r^2^ = 0.44), however SNP haplotypes can tag these variants efficiently (max *r*^*2*^ = 0.92) and are more strongly correlated with cholesterol levels than individual SNP [56]. High density genotyping of the HP/HPR locus will be required to understand the role of this locus in the response to trypanosome infection. Although rs8062041 is within *HP*, the known involvement of *HPR* in APOL1 mediated killing means that increased expression of *HPR* is another mechanism by which this SNP could be associated with the observed difference in likelihood of developing HAT. Our results showing an association between HAT and one SNP located within a CNV spanning *HP* and *HPR* duplication are not in line with results of Hardwick *et al*. [18] who observed no association with the *HPR* duplication allele and HAT in DRC. The difference between these results could be linked to the position of SNP within *HP*, the genetic diversity between the studied populations as well as the sampling methods. In our study, a case control approach was used while Hardwick *et al*. [18] used family-based sampling. Bresalier *et al*. [57] reported an association between polymorphisms at some HPR loci with an increasing risk of developing colon cancer. Similar associations were outlined by Tabak *et al*. [58] for *HPR*/*APOL1* loci variations in hepatoma and leukemia. There are also examples of CNV mediating different susceptibilities to infectious diseases [59, 60].

Of the twelve SNPs of *IL6* identified and investigated in our study, none of them revealed an association with HAT. However, with similar investigation on the same gene, Courtin *et al*. [15] showed a T allele of the *IL6* (4339) SNP rs2069849 which was significantly (Bonferroni corrected p = 0.04) associated with a decreased risk of developing HAT in the DRC. This SNP was not genotyped in this study because it could not be multiplexed with the others in the panel. The discrepancy between our results and those of Courtin *et al*. [15] could be due to insufficient linkage between our marker SNP and rs2069849 and or genetic differences between the DRC and Cameroon populations. The study designs also differed; we used a case control approach while Courtin *et al*. [15] used a family-based design and our study was smaller.

It has been suggested that the G1 and G2 alleles of APOL1 which increase the risk of developing kidney disease are under selection because they confer resistance to HAT [17]. Our observations on *APOL1* are consistent with Cooper *et al*. [22] who found no association with *APOL1* G1 and G2 in a comparison of cases and active *T*.*b*.*gambiense* HAT.

Concerning *HLA-G*, our results showed a protective effect of developing HAT for the loci rs17875389 G/A (p = 0.0416 and OR of 2.291) and an increased risk effect for rs1233330 A/G (p = 0.01105 and OR of 0.2754). These results support those of Courtin *et al*. [14] who reported similar results for different SNPs of the same genes in the DRC.

The association with *IL4* rs2070874 T/C (p = 0.00712 and OR of 0.6151) is the first time this has been observed in HAT although associations with IL4 have been observed in South American trypanosomiasis [61, 62]. The presence of IL4 in extravascular tissues promotes alternative activation of macrophages into M2 cells and inhibits classical activation of macrophages into M1 cells. This increase in repair macrophages (M2) is coupled with secretion of IL10 and TGFB that result in a diminution of pathological inflammation [63].

The results discussed above for *IL4* and *HLA-G* are based on FDR_BH values should be used with caution because no association was found after correction for multiple testing. However, we were only able to collect a relatively small number of cases (56) for this study, despite conducting large-scale field surveys. Whilst our power calculations indicated that effects of the sizes observed could be detected with our relatively small number of samples, larger cohorts of well phenotyped cases and controls may be required to confirm these observations. Therefore, although the present data is only suggestive of an association, the finding of suggestive associations in multiple populations increases the probability that these are genuine associations with disease [64]. This challenge is precisely what the TrypanoGEN network, a consortium of partners in eight African and three European countries seeks to address. The network has collected from seven regions in six countries (Cameroon, Cote d’Ivoire, DRC, Malawi, Uganda, and Zambia) a total of 3301 samples from cases and controls to include in a genome-wide-association study [44] which will be used to test the hypotheses generated here.

## Conclusion

The results of this study reveal an absence of association between HAT and several SNPs identified in genes previously associated with HAT within inhabitants of sleeping sickness foci of other African countries. An association between one SNP in *HP* and the susceptibility to HAT was revealed in inhabitants of sleeping sickness foci of Cameroon. Located within a CNV that spans both *HP* and *HPR* and given the known involvement of *HPR* in response to HAT, the association of rs8062041 with a CNV is the most plausible mechanism by which this SNP could be associated with protection against HAT. Our results reveal also that the association between host genetic determinants or gene polymorphisms and the susceptibility to *T*. *b*. *gambiense* infections may vary according to studied populations.

## Supporting information

S1 Fig Principal component analysis based on individual genotypes from the chronic HAT endemic area (foci) in Cameroon. (TIF)

S1 Table Candidate genes and SNPs identified and selected for this study. (DOCX)

S2 Table Fisher association analysis results. (DOCX)

S3 Table Fisher association of all loci genotype before quality control. (DOCX)
