# Supplementary figures and images for: A polymorphism in the haptoglobin, haptoglobin related protein locus is associated with risk of human sleeping sickness within Cameroonian populations
Source: PLoS Negl Trop Dis. 2017 Oct 27;11(10):e0005979. doi: 10.1371/journal.pntd.0005979 (PMC5697879; doi:10.1371/journal.pntd.0005979)

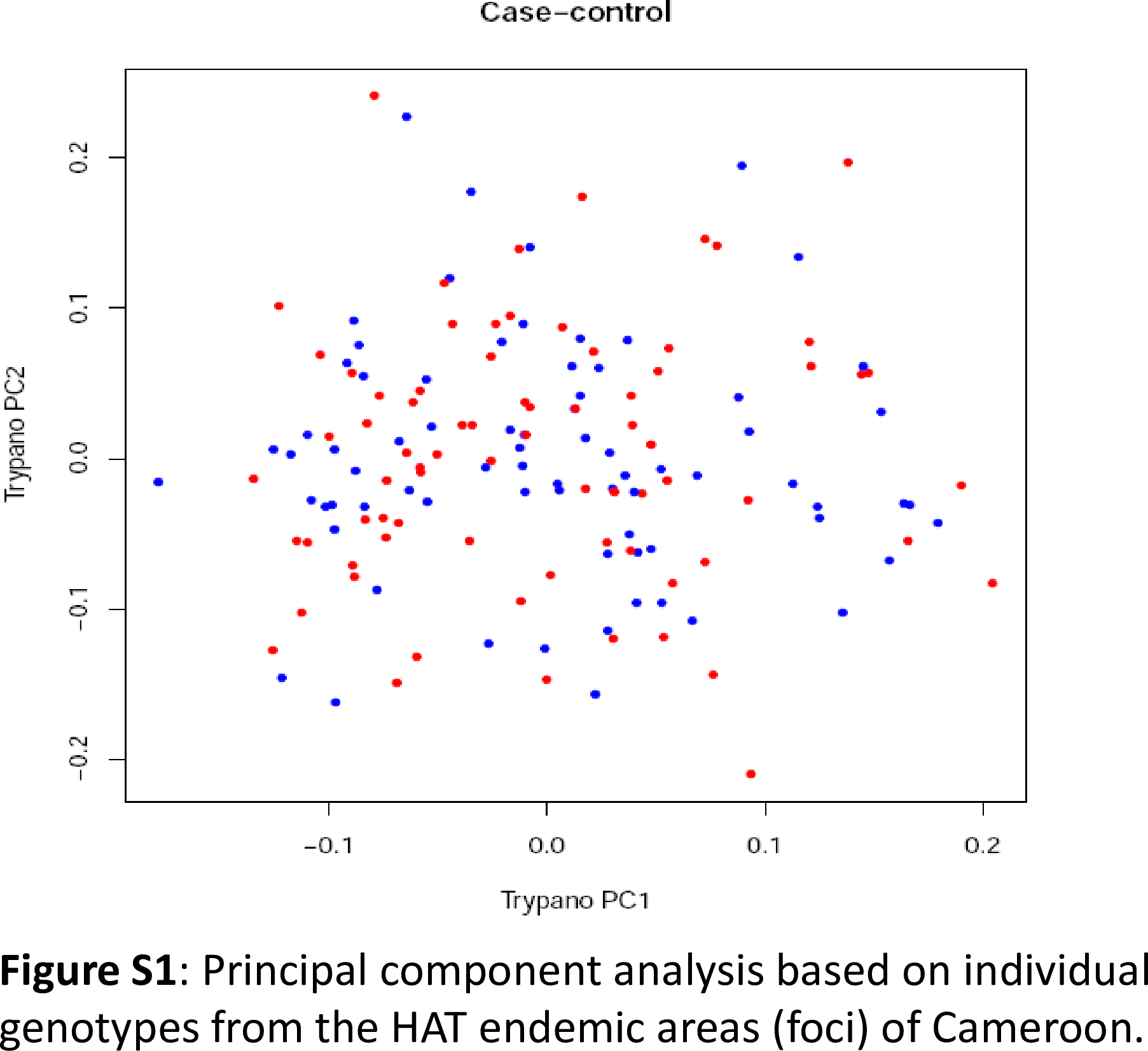

Supplement: S1 Fig — (TIF) [file pntd.0005979.s001.tif]
