# Supplementary material for: A polymorphism in the haptoglobin, haptoglobin related protein locus is associated with risk of human sleeping sickness within Cameroonian populations
Source: PLoS Negl Trop Dis. 2017 Oct 27;11(10):e0005979. doi: 10.1371/journal.pntd.0005979 (PMC5697879; doi:10.1371/journal.pntd.0005979)
Supplement: S2 Table — (DOCX) [file pntd.0005979.s003.docx]

**Table S2: Fisher association analysis results.**

| CHR | Gene | SNP | BP | F_A | F_U | Nominal P | OR | L95 | U95 | Hwe-P | Bonf | MAF | FDR-BH | F_ST_ |
| --- | --- | --- | --- | --- | --- | --- | --- | --- | --- | --- | --- | --- | --- | --- |
| 22 | APOL1 | rs73885319 | 36661906 | 0.1471 | 0.1313 | 0.6601 | 1.141 | 0.5745 | 2.264 | 1 | 1 | 0.1367 | 0.8423 | -0.0057 |
| 22 |  | rs136174 | 36661536 | 0.03191 | 0.08511 | 0.1043 | 0.3544 | 0.1006 | 1.248 | 1 | 1 | 0.06738 | 0.7904 | -0.0150 |
| 22 |  | rs73885316 | 36661674 | 0.03 | 0.025 | 0.8616 | 1.206 | 0.2824 | 5.152 | 1 | 1 | 0.02667 | 0.8252 | -0.0070 |
| 22 |  | rs136177 | 36661842 | 0.08163 | 0.1042 | 0.6041 | 0.7644 | 0.3239 | 1.804 | 0.5909 | 1 | 0.09655 | 0.8292 | -0.0040 |
| 22 |  | rs71785313 | 36662046 | 0.1078 | 0.1122 | 0.9232 | 0.956 | 0.444 | 2.058 | 0.09463 | 1 | 0.1107 | 0.92332 | -0.0080 |
| 6 | HLAG | rs1233330 | 29799103 | 0.04167 | 0.1364 | 0.01105 | 0.2754 | 0.0935 | 0.811 | 0.01562 | 0.6854 | 0.1054 | 0.3632 | 0.0305 |
| 6 |  | rs1233330 | 29799103 | 0.03922 | 0.12 | 0.01602 | 0.2993 | 0.1009 | 0.8875 | 0.6265 | 1 | 0.09272 | 0.3632 | 0.0371 |
| 6 |  | rs17875389 | 29794484 | 0.1176 | 0.055 | 0.04159 | 2.291 | 0.9735 | 5.391 | 1 | 1 | 0.07616 | 0.6708 | 0.0194 |
| 19 |  | rs1736936 | 298226540 | 0.4271 | 0.4579 | 0.6613 | 0.8825 | 0.3765 | 1.448 | 0.5359 | 1 | 0.4476 | 0.8423 | -0.0047 |
| 6 |  | rs12662618 | 29800211 | 0.2255 | 0.2041 | 0.6036 | 1.135 | 0.6358 | 2.028 | 0.5394 | 1 | 0.2114 | 0.8292 | -0.0066 |
| 6 |  | rs141206123 | 29799849 | 0.03922 | 0.03093 | 0.6219 | 1.279 | 0.3526 | 4.639 | 1 | 1 | 0.03378 | 0.8292 | -0.0063 |
| 6 |  | rs2517898 | 298799746 | 0.3646 | 0.3511 | 0.8452 | 1.064 | 0.6358 | 1.77 | 0.6501 | 1 | 0.3556 | 0.8952 | -0.0081 |
| 6 |  | rs1632932 | 29798039 | 0.4773 | 0.463 | 0.8431 | 1.059 | 0.6297 | 1.781 | 0.02537 | 1 | 0.468 | 0.8952 | -0.0055 |
| 6 |  | rs371194629 | 29798581 | 0.449 | 0.375 | 0.23057 | 0.4688 | 0.8288 | 2.225 | 7 0.469 0.2806 | 1 | 0.4 | 0.7904 | 0.0030 |
| 16 | HP | rs8062041 | 72088964 | 0.2959 | 0.4948 | **0.0002395** | **0.359** | 0.204 | 0.6319 | 1 | **0.01629** | 0.4281 | 0.01629 | 0.0987 |
| 16 |  | rs1424241 | 72078907 | 0.2188 | 0.1633 | 0.2283 | 1.435 | 0.7762 | 2.653 | 0.2732 | 1 | 0.1815 | 0.7904 | 0.0032 |
| 16 | HPR | rs2021171 | 72110541 | 0.3137 | 0.25 | 0.2471 | 1.371 | 0.8099 | 2.322 | 0.6029 | 1 | 0.2715 | 0.7904 | 0.0031 |
| 16 |  | rs7185840 | 72102112 | 0.1765 | 0.13 | 0.2654 | 1.434 | 0.7449 | 2.761 | 0.2081 | 1 | 0.1457 | 0.7904 | -0.0007 |
| 1 | IL10 | rs1800872 | 206946407 | 0.3333 | 0.38 | 0.4134 | 0.8158 | 0.4943 | 1.346 | 0.03289 | 1 | 0.3642 | 0.7904 | -0.0019 |
| 1 | CFH | rs1061170 | 196659237 | 0.3404 | 0.4176 | 0.2127 | 0.7197 | 0.426 | 1.216 | 0.456 | 1 | 0.3902 | 0.7904 | 0.0064 |
| 2 | IL1B | rs1143629 | 113593518 | 0.3889 | 0.4474 | 0.3349 | 0.7861 | 0.4715 | 1.311 | 0.8355 | 1 | 0.4286 | 0.7904 | -0.0016 |
| 16 | IL4R | rs1801275 | 27374400 | 0.2045 | 0.1582 | 0.3542 | 1.369 | 0.7184 | 2.608 | 1 | 1 | 0.1725 | 0.7904 | -0.0011 |
| 19 | IL12RB1 | rs11575934 | 18186618 | 0.09 | 0.1031 | 0.7596 | 0.8604 | 0.3765 | 1.966 | 1 | 1 | 0 | 0.8468 | -0.0076 |
| 5 | IL4 | rs2070874 | 132009710 | 0.3529 | 0.47 | 0.00712 | 0.6151 | 0.3761 | 1.006 | 0.5463 | 1 | 0.4305 | 0.6708 | 0.0207 |
| 5 |  | rs734244 | 132010726 | 0.2935 | 0.4091 | 0.05918 | 0.6 | 0.3529 | 1.02 | 0.1508 | 1 | 0.3724 | 0.6708 | 0.0160 |
| 5 |  | rs2243250 | 132009154 | 0.3235 | 0.2374 | 0.1143 | 1.537 | 0.9058 | 2.606 | 0.5778 | 1 | 0.2667 | 0.7904 | 0.0119 |
| 5 |  | rs2243270 | 132014109 | 0.2549 | 0.2222 | 0.0.519 | 1.197 | 0.6858 | 2.09 | 1 | 1 | 0.08607 | 0.8025 | -0.0045 |
| 5 |  | rs9282745 | 132014000 | 0.03922 | 0.07576 | 0.2675 | 0.498 | 0.1609 | 1.541 | 1 | 1 | 0.06333 | 0.7904 | 0.0043 |
| 5 |  | rs2243255 | 132011737 | 0.08 | 0.1209 | 0.2726 | 0.6324 | 0.2706 | 1.478 | 0.3499 | 1 | 0.1064 | 0.7904 | 0.0013 |
| 5 |  | rs2243268 | 132013963 | 0.2353 | 0.2929 | 0.3074 | 0.7427 | 0.4283 | 1.288 | 0.8098 | 1 | 0.02733 | 0.7904 | 0.0007 |
| 5 |  | rs2243261 | 132012806 | 0.16 | 0.1173 | 0.322 | 1.433 | 0.7192 | 2.854 | 0.3514 | 1 | 0.1318 | 0.7904 | 0.0006 |
| 5 |  | rs2243258 | 132012110 | 0.1078 | 0.08333 | 0.4628 | 1.33 | 0.5925 | 2.984 | 0.4938 | 1 | 0.09184 | 0.7954 | -0.0005 |
| 5 |  | rs2243279 | 132016227 | 0.1078 | 0.085 | 0.4679 | 1.301 | 0.5853 | 2.8931 | 0.5239 | 1 | 0.09272 | 0.7954 | -0.0054 |
| 5 |  | rs2243256 | 132011753 | 0.08824 | 0.105 | 0.6162 | 0.8249 | 0.3633 | 1.873 | 1 | 1 | 0.09934 | 0.8292 | 0.0062 |
| 5 |  | rs73269366 | 132018749 | 0.03922 | 0.055 | 0.6836 | 0.7013 | 0.2176 | 2.26 | 1 | 1 | 0.04967 | 0.8423 | -0.0044 |
| 5 |  | rs2243283 | 132016593 | 0.2347 | 0.25 | 0.8286 | 0.92 | 0.5182 | 1.633 | 0.5762 | 1 | 0.2447 | 0.8952 | -0.0078 |
| 7 | IL6 | rs1818879 | 22772727 | 0.05682 | 0.1062 | 0.2042 | 0.5067 | 0.3686 | 1.605 | 0.000182 | 1 | 0.08871 | 0.8025 | -0.0005 |
| 7 |  | rs2069845 | 22770149 | 0.2647 | 0.3333 | 0.2129 | 0.72 | 0.4238 | 1.223 | 1 | 1 | 0.31 | 0.7904 | 0.0029 |
| 7 |  | rs2069837 | 22768027 | 0.125 | 0.1667 | 0.3462 | 0.7143 | 0.3508 | 1.454 | 0.7285 | 1 | 0.1531 | 0.7904 | -0.0013 |
| 7 |  | rs2069830 | 22767137 | 0.08824 | 0.06061 | 0.4121 | 1.5 | 0.6102 | 3.687 | 0.03503 | 1 | 0.07 | 0.7904 | -0.0026 |
| 7 |  | rs2069842 | 22769310 | 0.06863 | 0.09596 | 0.4538 | 0.6942 | 0.2818 | 1.71 | 0.5935 | 1 | 0.08667 | 0.7954 | -0.0021 |
| 7 |  | rs2069855 | 22772624 | 0.05882 | 0.04211 | 0.4816 | 1.422 | 0.4795 | 4.216 | 1 | 1 | 0.04795 | 0.7987 | -0.0049 |
| 7 |  | rs2069843 | 22769994 | 0.09804 | 0.1263 | 0.5106 | 0.7522 | 0.3463 | 1.634 | 0.6472 | 1 | 0.1167 | 0.8025 | -0.0046 |
| 7 |  | rs2069834 | 22767828 | 0.05208 | 0.07576 | 0.5449 | 0.6703 | 0.2363 | 1.902 | 1 | 1 | 0.06803 | 0.8025 | -0.0028 |
| 7 |  | rs2066992 | 22768249 | 0.04082 | 0.05263 | 0.6716 | 0.766 | 0.2339 | 2.508 | 1 | 1 | 0.04861 | 0.8423 | -00059 |
| 7 |  | rs62449495 | 22764338 | 0.09091 | 0.07732 | 0.7308 | 1.193 | 0.4863 | 2.928 | 1 | 1 | 0.08156 | 0.8423 | -0.0064 |
| 4 | IL8 | rs13112910 | 74609755 | 0.35 | 0.2959 | 0.3231 | 1.281 | 0.7672 | 2.139 | 0.3302 | 1 | 0.3142 | 0.7904 | -0.0007 |
| 4 |  | rs2227545 | 74608727 | 0.07 | 0.06122 | 0.7115 | 1.154 | 0.4397 | 3.029 | 0.3009 | 1 | 0.06417 | 0.8423 | -0.0073 |
| 4 |  | rs58478511 | 74610033 | 0.2556 | 0.2809 | 0.7187 | 0.8788 | 0.4943 | 1.563 | 0.6037 | 1 | 0.2724 | 0.8423 | -0.0068 |
| 4 |  | rs2227307 | 74606669 | 0.47 | 0.4897 | 0.7591 | 0.9241 | 0.57 | 1.498 | 0.6872 | 1 | 0.483 | 0.8468 | -0.0064 |
| 4 |  | rs114259658 | 74605639 | 0.03 | 0.03571 | 0.8689 | 0.8351 | 0.2112 | 3.301 | 1 | 1 | 0.03378 | 0.8952 | -0.0069 |
| 12 | INFG | rs1861493 | 68551196 | 0.02273 | 0.07071 | 0.1299 | 0.3056 | 0.0679 | 1.375 | 1 | 1 | 0.05594 | 0.7904 | 0.0140 |
| 12 |  | rs78554979 | 68554636 | 0.1275 | 0.07653 | 0.1784 | 1.763 | 0.8041 | 3.8641 | 0.4396 | 1 | 0.09396 | 0.7904 | 0.0073 |
| 12 |  | rs2069705 | 68555011 | 0.3469 | 0.4286 | 0.1868 | 0.7083 | 0.4283 | 2.608 | 0.4137 | 1 | 0.04014 | 0.7904 | 0.0059 |
| 12 |  | rs2069718 | 68550162 | 0.43 | 0.355 | 0.1876 | 1.371 | 0.8393 | 2.238 | 0.2828 | 1 | 0.38 | 0.7904 | 0.0045 |
| 12 |  | rs2069728 | 68547784 | 0.36 | 0.3041 | 0.3256 | 1.287 | 0.7727 | 2.144 | 0.3404 | 1 | 0.3231 | 0.7904 | -0.0041 |
| 12 |  | rs2069723 | 68548594 | 0.03922 | 0.02 | 0.3619 | 2 | 0.4898 | 8.167 | 1 | 1 | 0.02649 | 0.7904 | -0.0020 |
| 12 |  | rs2069720 | 68549710 | 0.05 | 0.035 | 0.4457 | 1.451 | 0.4487 | 4.693 | 1 | 1 | 0.04 | 0.7954 | -0.0043 |
| 12 |  | rs2069713 | 68552476 | 0.00984 | 0.025 | 0.5386 | 0.3861 | 0.0445 | 3.35 | 1 | 1 | 0.01987 | 0.8025 | -0.0014 |
| 12 |  | rs2430561 | 68552522 | 0.18 | 0.1979 | 0.6975 | 0.8896 | 0.4778 | 1.656 | 0.349 | 1 | 0.1918 | 0.8423 | -0.0065 |
| 12 |  | rs20697222 | 68548953 | 0.01389 | 0.04225 | 0.328 | 1.09 | 0.377 | 2.704 | 1 | 1 | 0.02649 | 0.7904 | 0.0025 |
| 22 | MIF | rs12483859 | 24234807 | 0.46 | 0.3542 | 0.08928 | 1.553 | 0.9497 | 2.541 | 0.8233 | 1 | 0.3904 | 0.7904 | 0.0156 |
| 22 |  | rs35235644 | 24237822 | 0.1531 | 0.101 | 0.2185 | 1.608 | 0.7842 | 3.299 | 1 | 1 | 0.1182 | 0.7904 | 0.0053 |
| 22 |  | rs34383331 | 24238079 | 0.15 | 0.1212 | 0.4184 | 1.279 | 0.6383 | 2.564 | 0.3497 | 1 | 0.1309 | 0.7904 | -0.0042 |
| 6 | TNFA | rs1800629 | 31543031 | 0.1275 | 0.09694 | 0.3796 | 1.361 | 0.3485 | 2.633 | 0.5929 | 1 | 0.0604 | 0.7904 | -0.023 |
| 6 |  | rs1800630 | 31542476 | 0.05882 | 0.06122 | 0.8992 | 0.9583 | 0.6427 | 2.881 | 1 | 1 | 0.1074 | 0.9126 | -0.0080 |
| 5 | IL12B | rs2546890 | 15879900 | 0.3125 | 0.3469 | 0.5547 | 0.8550 | 0.5075 | 1.442 | 0.2688 | 1 | 0.3356 | 0.8025 | -0.0046 |
| 5 |  | rs3212227 | 158742950 | 0.4255 | 0.3776 | 0.4062 | 1.221 | 0.7403 | 2.0154 | 1 | 1 | 0.3931 | 0.7904 | -0.0028 |

SNP: single nucleotide polymorphism, BP base-pair location; F_A & F_U frequency of the minor allele in cases and controls respectively; Nominal P unadjusted asymptotic probability value; OR odds ratio; HWE-P Hardy-Weinberg equilibrium p value for unaffected individuals; BONF Bonferroni adjusted asymptotic p value, FDR_BH = False Discovery Rate Benjamin-Hochberg, MAF Minor allele frequency, CHR: Chromosome. The level of significance is 0.05.
