# Supplementary material for: A polymorphism in the haptoglobin, haptoglobin related protein locus is associated with risk of human sleeping sickness within Cameroonian populations
Source: PLoS Negl Trop Dis. 2017 Oct 27;11(10):e0005979. doi: 10.1371/journal.pntd.0005979 (PMC5697879; doi:10.1371/journal.pntd.0005979)
Supplement: S3 Table — (DOCX) [file pntd.0005979.s004.docx]

**Table S3: Fisher association of all loci genotype before quality control.**

| CHR | SNP and GENE | BP | Nominal P | OR | L95 | U95 | BONF |
| --- | --- | --- | --- | --- | --- | --- | --- |
| 1 | rs1061170_CFH | 196659237 | 0.1743 | 0.7079 | 0.4217 | 1.188 | 1 |
| 1 | rs1800872_IL10 | 206946407 | 0.6669 | 0.8857 | 0.5441 | 1.442 | 1 |
| 2 | rs1143629_IL1B | 113593518 | 0.407 | 0.7941 | 0.4783 | 1.319 | 1 |
| 4 | rs114259658_IL8 | 74605639 | 0.87 | 0.8182 | 0.207 | 3.233 | 1 |
| 4 | rs2227307_IL8 | 74606669 | 0.7606 | 0.9263 | 0.5732 | 1.497 | 1 |
| 4 | rs2227545_IL8 | 74608727 | 0.7138 | 1.107 | 0.422 | 2.902 | 1 |
| 4 | rs13112910_IL8 | 74609755 | 0.4727 | 1.207 | 0.7252 | 2.009 | 1 |
| 4 | rs58478511_IL8 | 74610033 | 0.615 | 0.8533 | 0.4806 | 1.515 | 1 |
| 4 | rs62312369_IL8 | 74610397 | 0.3326 | 0 | 0 | Nan | 1 |
| 5 | rs3212227_IL12B | 158742950 | 0.4128 | 1.236 | 0.7549 | 2.025 | 1 |
| 5 | rs2243250_IL4 | 132009154 | 0.0668 | 1.63 | 0.9668 | 2.747 | 1 |
| 5 | rs2070874_IL4 | 132009710 | 0.0446 | 0.597 | 0.3657 | 0.9747 | 1 |
| 5 | rs734244_IL4 | 132010726 | 0.05918 | 0.6 | 0.3529 | 1.02 | 1 |
| 5 | rs2243255_IL4 | 132011737 | 0.3782 | 0.7038 | 0.3111 | 1.593 | 1 |
| 5 | rs2243256_IL4 | 132011753 | 0.6164 | 0.8075 | 0.3558 | 1.833 | 1 |
| 5 | rs2243258_IL4 | 132012110 | 0.141 | 1.774 | 0.8399 | 3.748 | 1 |
| 5 | rs2243261_IL4 | 132012806 | 0.323 | 1.399 | 0.703 | 2.786 | 1 |
| 5 | rs71889624_IL4 | 132013430 | 0.2393 | 0.551 | 0.2142 | 1.418 | 1 |
| 5 | rs2243268_IL4 | 132013963 | 0.3839 | 0.7845 | 0.4581 | 1.343 | 1 |
| 5 | rs9282745_IL4 | 132014000 | 0.2707 | 0.488 | 0.1577 | 1.51 | 1 |
| 5 | rs2243270_IL4 | 132014109 | 0.4349 | 1.227 | 0.7068 | 2.131 | 1 |
| 5 | rs2243279_IL4 | 132016227 | 0.2707 | 1.505 | 0.7009 | 3.231 | 1 |
| 5 | rs2243282_IL4 | 132016554 | 0.07026 | 0.6226 | 0.3754 | 1.033 | 1 |
| 5 | rs2243283_IL4 | 132016593 | 0.7188 | 0.8961 | 0.5053 | 1.589 | 1 |
| 5 | rs2243285_IL4 | 132016993 | 0.5 | NA | NA | NA | 1 |
| 5 | rs73269366_IL4 | 132018749 | 0.8963 | 0.8506 | 0.2876 | 2.516 | 1 |
| 5 | rs2546890_IL12B | 158759900 | 0.56 | 0.8457 | 0.5049 | 1.417 | 1 |
| 6 | rs142798055_HLAG | 29793404 | 0.8403 | 0.7143 | 0.1222 | 4.175 | 1 |
| 6 | rs17875389_HLAG | 29794484 | 0.0551 | 2.193 | 0.9331 | 5.156 | 1 |
| 6 | rs1130355_HLAG | 29795993 | 0.5 | NA | NA | NA | 1 |
| 6 | rs17875406_HLAG | 29797448 | 0.5 | NA | NA | NA | 1 |
| 6 | rs1130363_HLAG | 29797696 | 0.4021 | 0.7949 | 0.4751 | 1.33 | 1 |
| 6 | rs1632932_HLAG | 29798039 | 0.8466 | 1.065 | 0.6403 | 1.772 | 1 |
| 6 | rs371194629_HLAG | 29798581 | 0.2398 | 0.2474 | 2.147 | 0.8288 | 1 |
| 6 | rs17179108_HLAG | 29798642 | 0.08354 | 1.898 | 0.878 | 4.102 | 1 |
| 6 | rs9380142_HLAG | 29798794 | 0.2112 | 1.385 | 0.831 | 2.307 | 1 |
| 6 | rs1610696_HLAG | 29798803 | 0.7041 | 0.8655 | 0.4706 | 1.592 | 1 |
| 6 | rs1233330_HLAG | 29799103 | 0.006542 | 0.2639 | 0.08967 | 0.7766 | 0.6411 |
| 6 | rs1611139_HLAG | 29799116 | 0.829 | 1.044 | 0.5929 | 1.838 | 1 |
| 6 | rs2517898_HLAG | 29799746 | 0.8468 | 1.04 | 0.6266 | 1.725 | 1 |
| 6 | rs141206123_HLAG | 29799849 | 0.6263 | 1.205 | 0.3325 | 4.368 | 1 |
| 6 | rs12661041_HLAG | 29800062 | 0.2213 | 0.7034 | 0.395 | 1.253 | 1 |
| 6 | rs2517897_HLAG | 29800101 | 0.5916 | 0.8444 | 0.4561 | 1.563 | 1 |
| 6 | rs12662618_HLAG | 29800211 | 0.4268 | 1.237 | 0.7053 | 2.168 | 1 |
| 6 | rs34783406_TNFA | 29832415 | 0.757 | 1.082 | 0.6647 | 1.76 | 1 |
| 6 | rs1136754_HLAA | 29911921 | 0.5 | NA | NA | NA | 1 |
| 6 | rs1059563_HLAA | 29911928 | 0.5 | NA | NA | NA | 1 |
| 6 | rs1059564_HLAA | 29911930 | 0.06074 | 6.155 | 0.632 | 59.94 | 1 |
| 6 | rs1800630_TNFA | 31542476 | 0.9009 | 0.902 | 0.3287 | 2.475 | 1 |
| 6 | rs1800629_TNFA | 31543031 | 0.4986 | 1.275 | 0.6032 | 2.694 | 1 |
| 7 | rs62449495_IL6 | 22764338 | 0.7308 | 1.193 | 0.4863 | 2.928 | 1 |
| 7 | rs2069830_IL6 | 22767137 | 0.4126 | 1.438 | 0.5856 | 3.532 | 1 |
| 7 | rs2069834_IL6 | 22767828 | 0.5449 | 0.6703 | 0.2363 | 1.902 | 1 |
| 7 | rs2069837_IL6 | 22768027 | 0.3477 | 0.6977 | 0.3429 | 1.419 | 1 |
| 7 | rs1474347_IL6 | 22768124 | 0.9218 | 1.029 | 0.4728 | 2.239 | 1 |
| 7 | rs2066992_IL6 | 22768249 | 0.6725 | 0.75 | 0.2292 | 2.455 | 1 |
| 7 | rs2069842_IL6 | 22769310 | 0.4538 | 0.6942 | 0.2818 | 1.71 | 1 |
| 7 | rs1548216_IL6 | 22769773 | 0.2194 | 0.6825 | 0.3771 | 1.235 | 1 |
| 7 | rs2069843_IL6 | 22769994 | 0.402 | 0.7061 | 0.3256 | 1.531 | 1 |
| 7 | rs2069845_IL6 | 22770149 | 0.1362 | 0.6667 | 0.3939 | 1.128 | 1 |
| 7 | rs2069855_IL6 | 22772624 | 0.4853 | 1.365 | 0.4607 | 4.045 | 1 |
| 7 | rs1818879_IL6 | 22772727 | 0.7955 | 0.9075 | 0.4557 | 1.807 | 1 |
| 12 | rs2069728_IFNG | 68547784 | 0.3322 | 1.264 | 0.7628 | 2.093 | 1 |
| 12 | rs2069723_IFNG | 68548594 | 0.06804 | 2.94 | 0.811 | 10.66 | 1 |
| 12 | rs2069722_IFNG | 68548953 | 0.3306 | 0.3105 | 0.03668 | 2.629 | 1 |
| 12 | rs2069720_IFNG | 68549710 | 0.4516 | 1.392 | 0.4309 | 4.5 | 1 |
| 12 | rs2069718_IFNG | 68550162 | 0.2911 | 1.281 | 0.7878 | 2.082 | 1 |
| 12 | rs1861493_IFNG | 68551196 | 0.1299 | 0.3056 | 0.06796 | 1.375 | 1 |
| 12 | rs2069713_IFNG | 68552476 | 0.5415 | 0.3786 | 0.04366 | 3.284 | 1 |
| 12 | rs2430561_IFNG | 68552522 | 0.6987 | 0.8684 | 0.4669 | 1.615 | 1 |
| 12 | rs78554979_IFNG | 68554636 | 0.1272 | 1.836 | 0.8499 | 3.967 | 1 |
| 12 | rs2069705_IFNG | 68555011 | 0.2929 | 0.759 | 0.4636 | 1.242 | 1 |
| 16 | rs1424241_HPR | 72078907 | 0.3837 | 1.329 | 0.7209 | 2.449 | 1 |
| 16 | rs152828_HPR | 72123886 | 0.6753 | 0 | 0 | Nan | 1 |
| 16 | rs1801275_IL4R | 27374400 | 0.1611 | 1.521 | 0.8117 | 2.849 | 1 |
| 16 | rs8062041_HPR | 72088964 | 0.0007481 | 0.3917 | 0.2255 | 0.6804 | 0.07332 |
| 16 | rs7185840_HPR | 72102112 | 0.2122 | 1.521 | 0.8045 | 2.875 | 1 |
| 16 | rs2021171_HPR | 72110541 | 0.3114 | 1.297 | 0.7683 | 2.19 | 1 |
| 19 | rs11575934_IL12RB1 | 18186618 | 0.6136 | 0.8072 | 0.3538 | 1.842 | 1 |
| 19 | rs1736936_HLAG | 29794317 | 0.4242 | 0.8287 | 0.5094 | 1.348 | 1 |
| 19 | rs375947_IL12RB1 | 18180451 | 0.5 | NA | NA | NA | 1 |
| 22 | rs12483859_MIF | 24234807 | 0.05433 | 1.625 | 0.9998 | 2.64 | 1 |
| 22 | rs36086171_MIF | 24235455 | 0.8229 | 1.072 | 0.597 | 1.927 | 1 |
| 22 | rs9282783_MIF | 24236359 | 0.1759 | 1.8 | 0.7076 | 4.579 | 1 |
| 22 | rs36070976_MIF | 24236864 | 0.5 | NA | NA | NA | 1 |
| 22 | rs11548056._MIF | 24237053 | 0.5 | NA | NA | NA | 1 |
| 22 | rs2070766_MIF | 24237221 | 0.5 | NA | NA | NA | 1 |
| 22 | rs35235644_MIF | 24237822 | 0.2185 | 1.608 | 0.7842 | 3.299 | 1 |
| 22 | rs2000466_MIF | 24237862 | 0.1749 | 1.439 | 0.8293 | 2.498 | 1 |
| 22 | rs34383331_MIF | 24238079 | 0.531 | 1.222 | 0.6105 | 2.445 | 1 |
| 22 | rs136174_APOL1 | 36661536 | 0.1062 | 0.3468 | 0.0985 | 1.221 | 1 |
| 22 | rs73885316_APOL1 | 36661674 | 0.86 | 1.158 | 0.2714 | 4.945 | 1 |
| 22 | rs136177_APOL1 | 36661842 | 0.4707 | 0.7319 | 0.3105 | 1.725 | 1 |
| 22 | rs73885319_APOL1 | 36661906 | 0.7935 | 1.09 | 0.55 | 2.162 | 1 |
| 22 | rs143830837_APOL1 | 36662042 | 0.5 | NA | NA | NA | 1 |
| 22 | rs71785313_APOL1_G2 | 36662046 | 0.9238 | 0.9355 | 0.4347 | 2.013 | 1 |

SNP: single nucleotide polymorphism, BP base-pair location; Nominal P unadjusted asymptotic probability value; OR odds ratio; BONF Bonferroni adjusted asymptotic p value. The level of significance is 0.05.
